# French Pregnancy Physical Activity Questionnaire Compared with an Accelerometer Cut Point to Classify Physical Activity among Pregnant Obese Women

**DOI:** 10.1371/journal.pone.0038818

**Published:** 2012-06-11

**Authors:** Nadia Chandonnet, Didier Saey, Natalie Alméras, Isabelle Marc

**Affiliations:** 1 Paediatrics Department, Research Center of the Centre Hospitalier Universitaire de Québec, Laval University, Quebec City, Quebec, Canada; 2 Rehabilitation Department, Faculty of Medicine, Laval University, Quebec City, Quebec, Canada; 3 Research Center of the Institut Universitaire de Cardiologie et de Pneumologie de Québec, Laval University, Quebec City, Quebec, Canada; University of Bath, United Kingdom

## Abstract

Given the high risk for inactivity during pregnancy in obese women, validated questionnaires for physical activity (PA) assessment in this specific population is required before evaluating the effect of PA on perinatal outcomes. No questionnaire was validated in pregnant obese women. The Pregnancy Physical Activity Questionnaire (PPAQ) has been designed based on activities reported during pregnancy and validated in pregnant women. We translated the PPAQ to French and assessed reliability and accuracy of this French version among pregnant obese women. In this cross-sectional study, pregnant obese women were evenly recruited at the end of each trimester of pregnancy. They completed the PPAQ twice, with an interval of 7 days in-between, to recall PA of the last three months. Between PPAQ assessments, participants wore an accelerometer (Actigraph GT1M) during 7 consecutive days. Fourty-nine (49) pregnant obese women (29.8±4.2 yrs, 34.7±5.1 kg.m^−2^) participated to the study. The intraclass correlation coefficients (ICCs) between the two PPAQ assessments were 0.90 for total activity, 0.86 for light and for moderate intensity, and 0.81 for vigorous intensity activities. It ranged from 0.59 for “*Transportation*” to 0.89 for “*Household and Caregiving*” activities. Spearman correlation coefficients (SCCs) between the PPAQ and the Matthews’ cut point used to classify an activity of moderate and above intensity were 0.50 for total activity, 0.25 for vigorous intensity and 0.40 for moderate intensity. The correlations between the PPAQ and the accelerometer counts were 0.58 for total activity, 0.39 for vigorous intensity and 0.49 for moderate intensity. The highest SCCs were for “*Occupation*” and “*Household and Caregiving*” activities. Comparisons with other standard cutpoints were presented in files S1, S2, S3, S4, S5, S6, S7. The PPAQ is reliable and moderately accurate for the measure of PA of various intensities and types among pregnant obese women.

## Introduction

The assessment of maternal physical activity (PA) during pregnancy is crucial due to the close relationship between the PA levels and the health status [1]. Physical inactivity in daily life during pregnancy might increase the risk of onset or progress of perinatal events such as gestational diabetes mellitus, preeclampsia and prematurity [2], [3]. The impact of PA or inactivity on neonatal issues such as foetal growth or birth weight is still debated [4], [5], [6], [7], [8].

Although there is no doubt that PA may be beneficial for obese women, most studies looking at the impact and the safety of PA during pregnancy underrepresent women with a higher risk profile such as the obese women [2], [4], [9], [10], [11]. To understand the relationship between PA and perinatal outcomes among pregnant obese women, it is important to accurately estimate the PA levels in this population. Due to the multiple PA patterns found during pregnancy, valid tools should be used to avoid measurement error. For instance, the pregnant obese women spend more time at lower intensity activities but may perceive them as moderate or vigorous [12]. Thus, reliability and accuracy of the tools used to measure PA have to be documented in pregnant obese population. To measure the impact of PA on maternal and neonatal outcomes or to address the safety, large sample sizes are required and tools must be easy to comply to. Furthermore, as the PA levels decrease across the pregnancy, the measurements need to be repeated in order to assess the impact of PA separately in early, mid and late pregnancy so a tool must be reliable and accurate across the trimesters of pregnancy. There are few questionnaires available for the evaluation of PA in pregnant women but none were validated in pregnant obese women [13], [14], [15], [16], [17].

The main objectives of this cross-sectional study were 1) to assess the reproducibility of the French version of the Pregnancy Physical Activity Questionnaire (PPAQ) in pregnant obese women and 2) to analyze and compare the data from this self-administered questionnaire with PA data objectively measured by accelerometry.

## Methods

### Ethics Statement

The study was approved by the Research Ethics Committees of the Centre Hospitalier Universitaire de Québec (CHUQ) and the Centre de Santé et de Services Sociaux de la Vieille-Capitale (CSSS-VC). Each participant read and signed a written consent form.

### Study Subjects

Pregnant women were recruited from the community via study announcements, pamphlets as well as from family practice and obstetrical clinics at the CHUQ hospitals and the Family Medicine Units in Quebec City (QC, Canada). Data collection spanned May 2009 to January 2011.

Women with a body mass index (BMI) >29.0 kg·m^−2^ were eligible for the study according to the criteria for obesity during pregnancy at the time of the study [18]. The other inclusion criteria were: ≥18 years of age, singleton pregnancies, and intention to deliver at a participating hospital. Women were excluded if they had pre-pregnancy diabetes, hypertension or renal failure, or if they had a PA contraindication at the time of the recruitment.

### Study Design

The participants were evenly recruited at the end of the first, second and third trimesters of pregnancy. At visit 1, a PA assessment of the last trimester (i.e. past three months) was self-administered using the French PPAQ. Following visit 1, the women received a portable accelerometer [GT1M] (ActiGraph LLC, Pensacola, FL, USA) and were instructed to wear the device continuously for 7 days and nights. At visit 2, one week after the first visit, a trained research assistant collected the data from the accelerometer records and the women were asked to complete the PPAQ for a second time.

### Measures

#### Physical activity assessment by the PPAQ

In term of accuracy and reproducibility, no questionnaire validated for assessing PA during the pregnancy definitively surpasses the others [13], [14], [15], [16], [17]. Among them, we have considered the PPAQ for its design, with the aim of measuring the PA during pregnancy, and for its development based on data collected among prenatal care patients [13]. The PPAQ provides a quantitative measure of a wide range of PA types and intensities, including sedentariness. This last point was important as the PA levels across pregnancy, especially in the population of pregnant obese women, are low.

It is a 33-questions self-administered questionnaire [13] which provides a comprehensive assessment of four domains of PA including “*Sports and Exercises*” (*n*=9), “*Household and Caregiving*” (*n*=16), “*Transportation*” (*n*=3) and “*Occupation*” (*n*=5). The PPAQ measures the frequency and the duration of the activities, and an intensity value is assigned to each activity. The activities can be analyzed by type, by intensity or for the total energy expenditure. The PPAQ was originally validated among a sample of 54 pregnant women using 7 days of accelerometer measurement [13]. The intraclass correlation coefficients (ICCs) were good with r=0.78 for total activity (≥ light), 0.82 for moderate intensity, 0.81 for vigorous intensity activities and ranged from 0.83 for “*Sports and Exercises*” to 0.93 for “*Occupation*”. The Spearman correlation coefficients (SCCs) between the PPAQ total activity score (≥ light intensity) and the accelerometer values of minutes per day spent at moderate and above intensity activities, classified with published count cut points, were r=0.08 (with the Freedson’s cut point [19]), 0.32 (with the Swartz’s cut point [20]) and 0.43 (with the Hendelman’s cut point [21]). The correlations for the vigorous intensity (0.37) and the sports activities (0.48) were the highest when the questionnaire data were compared with the Actigraph counts (average counts per minute) [13]. A Japanese [22] and a Vietnamese [23] versions are available but both are not actually validated by a comparison with an accelerometer. Although the PPAQ seems to provide a reasonable measure of PA during pregnancy, additional information about the specificities and performances of this questionnaire were needed before assessing PA across the pregnancy, especially in a population of pregnant obese women.

For the purpose of this study, the PPAQ was translated to French, and tested for the acceptability of the wording (*n*=10). At the end of the “*Sports and Exercises*” section (questions #30 and 31), the women had the opportunity to report any unlisted activities in an open-ended section. In particular, they were able to report additional information on the practice of winter outdoor activities (e.g. skiing, snowshoeing). To account for the specificities related to the climate and the activities in Canada, one question related to outdoor chores (# 19) has been modified to include a winter outdoor activity according to its intensity (i.e. “shoveling snow”). That version is available in (See File S1) and the English version is included in the original development and validation study [13].

The PPAQ was self-administered and took about 10 min to complete. The reported time spent at each activity was multiplied by its intensity to obtain a weekly average of energy expenditure (MET-h.wk^−1^) attributable to each activity (where 1 MET is the metabolic equivalent of the energy expended at rest) and summed to derive the weekly total activity score. The average energy expended was also calculated according to each domains of activity and each intensity level (sedentary [<2.0 METs], light [2.0≤ activity <3.0 METs], moderate [3.0≤ activity ≤6.0 METs] or vigorous [>6.0 METs]) [24].

#### Accelerometer measurements

The accelerometers provide an objective measure of PA over an extended period of time. The reliability and validity of accelerometers have been examined extensively [25], [26], [27]. The Actigraph GT1M accelerometer, used to measure PA in this study, was not validated against the doubly-labeled water (DLW) criterion. However, in a comparison study of the GT1M with the Actigraph CSA-7164, which was validated against DLW, it was reported that the counts were slightly but significantly higher in the GT1M output. Nevertheless, the monitor was found to be accurate for the walking activities [28].

The GT1M Actigraph activity monitor is a biaxial accelerometer detecting the normal human movement (acceleration) while filtering out the high-frequency movements (e.g. vibration). The small accelerometer (3.8×3.7×1.8 cm; weight: 27 g) was worn on the right hip with an adjustable belt [29]. The women were instructed to wear the accelerometer 24 h.d^−1^ (i.e. all the time) for 7 consecutive days and nights. They were given a daily log to note the hours of sleep and whether they removed the accelerometer at any time (for bathing, swimming, convenience or comfort) or whether they practised activities that are not detected by accelerometers such as stationary bicycle. The GT1M accelerometer was initialized and the data downloaded according to the manufacturer’s specifications using the software (Actilife) provided by the company. The steps per day were automatically measured by a pedometer included in the device. The average total counts were defined as the mean vertical accelerometer’s output by 24 h period, reflecting the output without any categorization according to the intensity. According to the protocol, the days when the accelerometer was not worn for ≥8 h during the waking hours (i.e. excluding night) were excluded. The number of minutes per 24 h period (from 00h00 to 23h59 on a given day) spent at moderate and above intensity activities was calculated using the Matthews’ cut point (760 counts.min^−1^) [30]. Albeit we also performed comparison with other standard references to classify the accelerometer data (e.g. Freedson’s [19], Swartz’s [20] and Hendelman’s [21] cut points respectively at 1952, 574 and 191 counts.min^−1^), the Matthews’ one has the advantage to have not been obtained with a single linear regression equation and was developed by using calibration data of various population samples which included locomotion and lifestyle-based activities that are performed by pregnant women. In contrast, although they also derived from locomotion and lifestyle-based activities, the Swartz’s and the Hendelman’s cut points were obtained from linear regression equations. The Freedson’s cut point was derived only from locomotion activities, which are not representative of the PPAQ measurements.

#### Other measurements

The women’s height was measured at inclusion with a stadiometer, and the BMI prior to the pregnancy was calculated with the self-reported pre-pregnancy weight using a standardized question, as recommended by IOM [31]. The estimation of the pre-pregnancy BMI served to classify the pregnant women as obese [32]. The gestational age was based on the last menstruation period or on a first-trimester ultrasound measurement. The participants’ socio-demographic characteristics, lifestyle habits and obstetrical history were assessed by a questionnaire.

### Statistical and Data Analyses

Descriptive statistics were used to document the socio-demographic characteristics and lifestyle habits (e.g. smoking, alcohol consumption). For the categorical variables (e.g. marital status, race, parity, employment, smoking), the frequency distributions were calculated. For the continuous or ordinal measurements (e.g. maternal age, gestational age, GT1M measurements, PPAQ scores), the central location, means and variations (SD), were calculated. *P*-values for the comparison of the three trimesters were assessed with Kruskal-Wallis for continuous variables and the Fisher’s exact test for categorical variables.

The reproducibility of the PPAQ was evaluated by ICCs for total score and sub-scores according to the type and the intensity categories. To evaluate the accuracy of the questionnaire, the SCCs were calculated between the PPAQ completed at the first visit and the average Actigraph counts and total daily minutes obtained according to the classification of the intensity from the Matthews’ cut point mentioned above. Although the energy expenditure can be calculated from the accelerometer, the equations available have not been validated in pregnant women so we preferred not to use them to assess the accuracy.

Furthermore, the subjects were separated into tertiles according to their total energy expenditure as reported in the PPAQ. For each tertile, the means and variations (SD) were calculated from the accelerometer data. The Jonckheere-Terpstra test was performed on the tertiles to assess the trend. The validation of the French PPAQ was established using the average total daily minutes spent at moderate and above intensity activities, according to the Matthews’ cut point [30], but for information and comparison with the original validation study [13], the analyses were also performed using the Freedson’s, Swartz’s and Hendelman’s cut points (See File S2, File S3 and File S4). Moreover, all GT1M analyses (i.e. descriptive statistics, accuracy of the PPAQ and trend for tertiles of energy expenditure) were performed using the average daily minutes cumulated in bouts of at least 10 consecutive minutes spent at moderate and above intensity activities, according to the four previous cut points (See File S5, File S6 and File S7). All the results were considered significant with *P*-values ≤0.05 and all analyses were performed using SAS version 9.2 (SAS Institute Inc., Cary, NC, USA).

## Results

### Study Population

Fifty-six (56) participants were recruited for the study. Among them, 7 women were excluded from all the analyses (6 experienced technical problems with the accelerometers and one received a formal prescription for strict bed rest the day after her inclusion in the study). Therefore, accelerometer data were obtained for 49 women evenly distributed across the three trimesters of pregnancy. Among them, one participant was excluded from the accelerometry analyses because her daily log was not appropriately completed, leading to improper estimates of her wearing compliance [33]. However, the compliance to the wearing instructions of the accelerometer, including the completion of the daily log, was excellent since accelerometer data were available from at least 6 days for 47 (96%) women. In addition, the accelerometer was worn for a mean of 22.4±2.03 hours per day (14.4±1.05 hours per day during waking time).

The women characteristics were similar among the three trimesters in terms of age, pre-pregnancy BMI, and smoking during the past trimester (Table 1). The proportion of participants with an active work occupation decreased across the trimesters.

**Table 1:** Women’s characteristics.

		All participants (*n*=49)	First trimester (*n*=17)	Second trimester (*n*=16)	Third trimester (*n*=16)
		Mean ± SD or *n* (%)	Mean ± SD or *n* (%)	Mean ± SD or *n* (%)	Mean ± SD or *n* (%)
Age, year		29.8±4.2	28.5±4.8	30.8±4.3	30.3±3.3
Gestational age, week		24 5/7±9 0/7	13 6/7±6/7*	25 4/7±5/7*	35 4/7±6/7*
Pre-pregnancy BMI, kg.m^−2^		34.7±5.1	34.1±4.6	33.8±4.9	36.3±5.7
Married or living with a partner		48 (98%)	17 (100%)	16 (100%)	15 (94%)
White		49 (100%)	17 (100%)	16 (100%)	16 (100%)
Parity					
	0	21 (43%)	5 (29%)	8 (50%)	8 (50%)
	≥1	28 (57%)	12 (71%)	8 (50%)	8 (50%)
Schooling					
	High school or less	9 (18%)	4 (24%)*	5 (31%)*	0 (0%)*
	College/graduate	40 (82%)	13 (76%)	11 (69%)	16 (100%)
Employed during past trimester		20 (41%)	11 (65%)*	5 (31%)*	4 (25%)*
Smoking during past trimester		7 (14%)	3 (18%)	4 (25%)	0 (0%)
# alcohol consumptions† per wk during past trimester		0.21±0.46	0.35±0.69	0.15±0.26	0.12±0.24

* *P*<0.05.

† One consumption corresponds to 125 ml of wine, 350 ml of beer or 30 ml of spirit.

### Accelerometry

Women walked an average of 5259±1762 steps.d^−1^ during the pregnancy (Table 2). The average total daily minutes spent at moderate and vigorous intensity activities was 83±35 min.d^−1^ according to the Matthews’ cut point (see File S2 for the results using the Hendelman’s, Swartz’s and Freedson’s cut points). It was importantly lowered (17±16 min.d^−1^) when only the time cumulated in bouts of at least 10 consecutive minutes over the Matthews’ cut point was used (See File S5).

**Table 2:** Physical activity distribution during pregnancy from Actigraph’s GT1M recording.

			All participants (*n*=48)			First trimester (*n*=17)		Second trimester (*n*=16)	Third trimester (*n*=15)
			Mean ± SDor *n* (%)	Median	25^th^–75^th^percentile	Mean ± SDor *n* (%)		Mean ± SDor *n* (%)	Mean ± SDor *n* (%)
Counts (*n* ×10^4^.24 h^−1^)			20.1±6.7	19.1	15.5–26.2	21.3±5.8		19.4±6.3	19.5±8.1
Steps (.24 h^−1^)			5259±1762	5234	3728–6471	5719±1728		5001±1653	5014±1921
Moderate intensity or above (min.24 h^−1^)									
	Matthews’ cut point		83±35	72	59–106		88±25	78±36	83±43
Cumulating 150 min of moderate intensity activity by week									
		Matthews’ cut point	48 (100%)				17 (100%)	16 (100%)	15 (100%)

The diaries completed during the accelerometer wearing week revealed that 38.8% (*n*=19) of the participants reported, in total, 36 moments in which they performed an activity which is presumed to be underestimated by accelerometry (such as bicycle, weight lifting or stretching) or which is not measured at all (such as pool [sitting and swimming] or water calisthenics). Regardless of the intensity, the mean time per day spent at unmeasured or underestimated activities, for those women who did report such activities, was about 22 min.d^−1^.

### Physical Activity Self-reported by the PPAQ

The data from the PPAQ indicated that, overall, about 36±19% of the total energy expenditure (MET-h.wk^−1^) was reported to be spent at sedentary activities (Table 3). Almost half of the energy was related to “*Household and Caregiving*” activities (45±20%). By comparison, the energy spent at “*Sports and Exercises*” was very low (7±5%).

**Table 3:** Physical activity levels as self-reported by the PPAQ.

		All participants (*n*=49)				First trimester (*n*=17)		Second trimester (*n*=16)		Third trimester (*n*=16)	
		Mean ± SD	%	Median	25^th^–75^th^ percentile	Mean ± SD	%	Mean ± SD	%	Mean ± SD	%
Total energy expenditure, *METS•h.wk* ^−*1*^		202±82		180	132–269	234±84		186±86		185±68	
Energy expenditure in moderate activity andabove, *METS•h.wk* ^−*1*^		57±51		36	18–96	69±59		48±42		54±49	
Cumulating ≥10 *METS•h/wk* of Sports/Exercises (*n*[%])		26 (53%)				8 (47%)		8 (50%)		10 (63%)	
Total energy expenditure by intensity,*METS•h.wk* ^−*1*^											
	Sedentary	64±27	36±19	60	40–92	76±23	37±17	60±24	37±18	55±31	35±24
	Light	81±44	39±13	73	48–106	90±43	37±11	78±49	41±13	76±39	40±14
	Moderate	53±47	23±13	34	18–81	63±57	24±14	46±41	21±11	49±43	23±14
	Vigorous	4±9	1±3	0	0–2	5±12	2±4	2±2	1±1	4±9	2±3
Total energy expenditure by type, *METS•h.wk* ^−*1*^											
	Household/Caregiving	100±74	45±20	74	44–153	104±77	41±17	95±78	46±21	99±71	49±23
	Occupational	32±42	17±21	0	0–71	56±49*	26±21	22±35*	14±22	16±30*	10±19
	Sports/Exercises	14±13	7±5	11	4–20	14±16	5±5	13±11	7±6	15±14	7±5
	Transportation	17±11	9±5	14	8–23	23±9*	10±4*	15±12*	9±7*	13±7*	7±3*

* *P*<0.05.

### PPAQ Reproducibility

As measured by a test-retest with a one-week interval, the reproducibility, reported by ICCs between the two PPAQ assessments, was high (ICC=0.90 for total activity on pooled data [49 women]) (Table 4). The reproducibility was the lowest for the vigorous intensity activities (0.81) and ranged from 0.86 to 0.88 for sedentariness, light and moderate intensity activities. Among the types of activities, “*Transportation*” had the lowest ICC (0.59). ICCs were high for “*Household and Caregiving*” (0.89), “*Occupation*” (0.84) and “*Sports and Exercises*” (0.82).

**Table 4:** Reliability of the PPAQ.

Methods		
	1 week test-retest Consistency of estimates for total activity, activity type, and intensity (Intraclass correlation coefficients [ICCs])	
Sample		
	49 pregnant obese women aged 29.8±4.2, mean gestational age 24 5/7±9 0/7 wk, mean prepregnancy BMI 34.7±5.1 kg.m^−1^, 43% primiparous.	
Summary Results		
	Total activity (light and above)	0.90
	Sedentary (<2.0 METs)	0.88
	Light (2.0–<3.0 METs)	0.86
	Moderate (3.0–6.0 METs)	0.86
	Vigorous (>6.0 METs)	0.81
	Household/Caregiving	0.89
	Occupational (*n*=20)*	0.84
	Sports/Exercises	0.82
	Transportation	0.59

* Including only women who were still working in the past trimester.

### PPAQ Accuracy

To assess the accuracy of the PPAQ at different intensities and field settings, the summary measurements of the PPAQ were compared to the mean total counts per day and to the mean total number of minutes per day spent over the Matthews’ cut point (Table 5). As no major difference was observed between the trimesters, only the results on all participants pooled are reported. The SCCs between the PPAQ and the Matthews’ accelerometer cut point were 0.50 for total activity (light and above intensity), 0.25 for vigorous and 0.40 for moderate intensity activities reported in the PPAQ. The correlations for the types of activities ranged from 0.27 for “*Sports and Exercises*” to 0.53 for “*Occupational*” activities. The mean values of counts per day correlated with the total energy expenditure as well as with most of the intensities and types of activities (Table 5. See File S3 for the results using the Hendelman’s, Swartz’s and Freedson’s cut points). The correlations for the average daily minutes cumulated in bouts of at least 10 consecutive minutes over the Matthews’ cut point were slightly lower than those with the total daily minutes (See File S6).

**Table 5:** Accuracy of the PPAQ.

Methods			
	Relationships between activity and Actigraph GT1M (criterion) data (Spearman correlation coefficients [SCCs])		
Summary Results			
	**PPAQ measures**	**Mean counts.d^−1^**	**Matthews’ cut point**
	Total activity (light and above)	**0.58 (** ***P*** **<0.01)**	**0.50 (** ***P*** **<0.01)**
	Sedentary (<2.0 METs)	−0.19 (*P*=0.19)	−0.17 (*P*=0.24)
	Light (2.0–<3.0 METs)	**0.53 (** ***P*** **<0.01)**	**0.46 (** ***P*** **<0.01)**
	Moderate (3.0–6.0 METs)	**0.49 (** ***P*** **<0.01)**	**0.40 (** ***P*** **<0.01)**
	Vigorous (>6.0 METs)	**0.39 (** ***P*** **<0.01)**	0.25 (*P*=0.08)
	Household/Caregiving	**0.56 (** ***P*** **<0.01)**	**0.48 (** ***P*** **<0.01)**
	Occupational (*n*=19)*	**0.56 (** ***P*** **=0.01)**	**0.53 (** ***P*** **=0.02)**
	Sports/Exercises	**0.40 (** ***P*** **<0.01)**	0.27 (*P*=0.06)
	Transportation	**0.38 (** ***P*** **<0.01)**	**0.29 (** ***P*** **<0.05)**

* Including only women who were still working in the past trimester.

Finally, the participants were separated into tertiles according to the total energy expenditure calculated from the PPAQ. Then, the mean total minutes per day, as measured by the Matthews’ accelerometer cut point, and the average total counts per 24 h period, have been derived for each tertile. A positive trend was observed across the tertiles for both accelerometers’ measures (Table 6. See File S4 for the results using the Hendelman’s, Swartz’s and Freedson’s cut points). Such association was not significant when using the average daily minutes cumulated in bouts of at least 10 consecutive minutes over the Matthew’s cut point (See File S7).

**Table 6:** Mean (SD) GT1M values across tertiles of total energy expenditure based on the PPAQ.

Actigraph measures	Lowest Tertile	Middle Tertile	Highest Tertile	Trend *P* *
	Mean ±SD (*n*=16)	Mean ±SD (*n*=17)	Mean ±SD (*n*=16)	
Counts (*n* x 10^4^.24 h^−1^)	17.4±5.3	18.7±6.1	24.4±6.8	**<0.01**
Matthews’ cut point	72±29	78±29	101±40	**0.04**

* Jonckheere-Terpstra.

## Discussion

This study investigated for the first time a French version of the PPAQ and indicated that it was highly reliable in pregnant obese women. Furthermore, using a semi-quantitative questionnaire, this cross-sectional study indicated that the levels of PA were low in pregnant obese women. Finally, a correlation between the data from the PPAQ and from the accelerometer confirmed a moderate but acceptable accuracy of the questionnaire in this population. The translation to diverse languages and the application of the PPAQ to specific sub-populations would provide data to improve the actual or design other questionnaires that assess PA in pregnant women with a better accuracy. This study provided information on the capacity of the questionnaire and the GT1M to measure the PA levels. More studies are needed to determine which level of PA might improve the perinatal outcomes.

The recent health strategies encourage 30 minutes per day of moderate intensity activities on almost every days of the week in pregnant women [34]. There is a need for accurate assessment techniques to measure a lower intensity PA. A growing body of literature recommends that questionnaires should assess PA, including not only the assessment of sports and recreational activities but also a full range of physical activities related to work, transportation and childcare as well as the assessment of sleep and inactivity time [35], [36].

Using the Matthews’ cut point, the accuracy of the French PPAQ for assessing the PA in pregnant obese women is moderate. Although the original English PPAQ validation study among pregnant women did not provide analyses using the Matthews’ cut point [13], the SCCs using the Freedson’s, Swartz’s and Hendelman’s cut points reported in that study are very close from those observed in pregnant obese women (See File S3). The SCCs using the Swartz’s and the Matthews’ cut points followed a similar pattern in pregnant obese women. Comparing to the Swartz’s cut point (the nearest cut point from the Matthews’ one), SCCs were slightly higher for total activity (≥ light) (0.56 in obese women versus 0.32 in pregnant women of any BMI), for light intensity activities (0.54 versus 0.10 respectively), for “*Household and Caregiving*” activities (0.55 versus −0.01 respectively) and for “*Occupational*” activities (0.61 versus 0.31 respectively). The validation of the PPAQ in this specific population is a significant contribution taking into account that PA in pregnant obese women is of high importance as it may decrease the occurrence of adverse outcomes related to obesity.

Although it may not represent the real absolute PA intensity levels in pregnant women, the estimations of time spent at moderate and above intensity activities were calculated from the accelerometer using the Matthews’ cut point [30] and compared to the energy expenditure levels based on the self-reported information from the PPAQ. The time spent at moderate-to-vigorous intensity activities, based on accelerometry, is surprisingly high. However, the accelerometer data analyses based on the time per day spent at moderate and above intensity activities in bouts of 10 consecutive minutes for each cut points is significantly reduced and is probably a more realistic description of the PA levels in this population of pregnant obese women (See File S5). As a matter of fact, the total daily minutes spent over an accelerometer threshold does not provide information on the distribution (consecutive minutes or not) of these activities over the day and the PA guidelines recommend that bouts of at least 10 consecutive minutes of moderate intensity physical activity could be cumulated to reach the daily 30 minutes recommended [37]. As health benefits from PA may depend on these distribution patterns, it may be interesting to determine which of these parameters (counts, total minutes or bouts of consecutive minutes) are associated to the healthiest outcomes during pregnancy.

There is no cut point developed and validated in pregnant women. Among the regression equations that were used in this study, two derived from locomotion and lifestyle-based activities [20], [21] and another from purely locomotion activities [19]. In fact, the first two equations (Swartz and Hendelman) overestimate the energy cost from low intensity activities as their intercept is high. Especially, it has been documented that the Hendelman’s equation should not be used as it importantly overestimates the time spent at moderate intensity activities. Conversely, the Freedson’s value underestimates the total daily energy expenditure and PA because its cut-off was calculated with high movement-to-energy expenditure ratio [38]. As this cut point [19] was derived from walking and running, it was not a suitable criterion for the validation of a free-living PA questionnaire. Moreover, the use of linear regression created in laboratory or in field settings with specific activities appears to overestimate the time spent at moderate intensity activities when applied to a free-living measurement [30]. An interesting alternative approach proposed by Matthews determined a threshold by combining data from studies conducted in various settings (field and laboratory). The 25^th^ percentile count value from six moderate intensity activities and the 75^th^ percentile from six light intensity activities were defined as the lower range of count value for moderate and the upper range for light intensity respectively. Intermediates values were tested in a new set of data and the 760 counts.min^−1^ cut-off was defined as a moderate-to-vigorous physical activity and found accurate [30]. This combination approach was confirmed to be an optimal balance between lifestyle and treadmill settings [38].

As mentioned previously, only few questionnaires are available for the measurement of PA during pregnancy [13], [14], [15], [16], [17]. Among them, the Kaiser Physical Activity Survey (KPAS) was validated in non-pregnant [39] and pregnant women [14] showing a good reliability (ICC of 0.84 for total activity) and a reasonable accuracy for the total activity compared with the counts (0.52) and other cut points. Comparable with the PPAQ in term of activities, this questionnaire only provides a score between 1 to 5 without quantifying the PA and leaving the comparison with other questionnaires more difficult. The Pregnancy Infection and Nutrition Physical Activity Questionnaire (PINPAQ) is similar to the PPAQ (i.e. not limited in term of activity intensities or types) but with open-ended questions only, resulting in lower SCCs (0.23 for total activity and 0.24 for moderate-to-vigorous intensity activities versus accelerometer counts) [16]. Other questionnaires showed no or weaker correlation or assessed only the moderate and vigorous intensity activities compared with an accelerometer during the pregnancy, such as the International Physical Activity Questionnaire (IPAQ) with a correlation of 0.15 for total activity in METs calculated from the accelerometer [15] and the Physical Activity and Pregnancy Questionnaire (PAPQ) with SCCs of 0.59 and 0.15 for vigorous and moderate intensity activities in min.wk^−1^ respectively [17]. No questionnaire was specifically validated in a population of pregnant obese women.

Some limitations are inherent in the present study. Both PPAQ and accelerometer errors might have affected the correlations. Although the percentage of sedentariness/light intensity activities remained very high, the PPAQ data might have been slightly inaccurate, with obese women reporting more activities than practised. Furthermore, the SCCs may be lowered by a decrease in the women’s activity across the pregnancy trimesters. The SCCs might also have been affected by the difference in the measurement times of the questionnaire and the accelerometer. The PA levels from the questionnaire, self-reported as PA usually performed per day or per week during the past 3 months, might have been overestimated by the participants in comparison with the PA levels measured by accelerometer during a week at the end of the 3-months period. Nevertheless, we felt that the limited and homogeneous levels of PA in pregnant obese women, with a large proportion of the time spent at sedentary or low intensity activities, were mainly responsible for the low-to-moderate SCCs of our study. The accelerometer error might be related to the failure of the Actigraph to measure the upper body movements, stationary bicycle and water activities. As documented by the diaries, and even if all activities were not of high intensity (such as pool or yoga), a failure of measurement by the accelerometer was observed. It justifies the use of the short diary in addition to accelerometry to document these activities. Finally, we cannot exclude a selection bias as it is possible that women who participated to the study were more interested by health and PA than the general population of pregnant obese women is.

This study highlights the challenge of quantifying PA using questionnaires or accelerometry alone. In fact, questionnaires and accelerometers are complementary tools to document the levels of PA as well as its impact on the pregnancy and neonatal outcomes in the pregnant obese population. The comparison between the PPAQ and accelerometry was a cornerstone before using these tools to assess the compliance to the PA recommendations in pregnant obese women.

We conclude that the French version of the PPAQ is reliable and reasonably accurate for the measure of PA of various intensities and types among pregnant obese women. For research requiring a detailed assessment of PA, both questionnaire and accelerometer should be used. Considering the high percentage of sedentary activities in pregnant obese women, accelerometer cut points defined by various types of activities (i.e. locomotion and lifestyle-based) and settings (i.e. free-living and laboratory), such as the one proposed by Matthews, may be used to evaluate the adherence to PA recommendations and the relative impact of a PA intervention.

## Supporting Information

File S1 **Questionnaire Français d’Activité Physique pendant la Grossesse.**
(PDF)

File S2 **Physical activity distribution during pregnancy from Actigraph’s GT1M recording in pregnant obese women (Hendelman’s, Swartz’s and Freedson’s cut points).**
(PDF)

File S3 **Accuracy of the French PPAQ in pregnant obese women (Hendelman’s, Swartz’s and Freedson’s cut points).**
(PDF)

File S4 **GT1M values across tertiles of total energy expenditure based on the French PPAQ in pregnant obese women (Hendelman’s, Swartz’s and Freedson’s cut points).**
(PDF)

File S5 **Physical activity distribution during pregnancy from Actigraph’s GT1M recording in pregnant obese women (Bouts of at least 10 consecutive minutes over standard cut points).**
(PDF)

File S6 **Accuracy of the French PPAQ in pregnant obese women (Bouts of at least 10 consecutive minutes over standard cut points).**
(PDF)

File S7 **GT1M values across tertiles of total energy expenditure based on the French PPAQ in pregnant obese women (Bouts of at least 10 consecutive minutes over standard cut points).**
(PDF)
