# Supplementary material for: French Pregnancy Physical Activity Questionnaire Compared with an Accelerometer Cut Point to Classify Physical Activity among Pregnant Obese Women
Source: PLoS One. 2012 Jun 11;7(6):e38818. doi: 10.1371/journal.pone.0038818 (PMC3372468; doi:10.1371/journal.pone.0038818)
Supplement: File S1 — Questionnaire Français d’Activité Physique pendant la Grossesse. (PDF) [file pone.0038818.s001.pdf]

Questionnaire Français d'Activité Physique pendant la Grossesse

Version française du Pregnancy Physical Activity Questionnaire [PPAQ]

Chandonnet N, Saey D, Alméras N, Marc I. French Pregnancy Physical Activity Questionnaire Compared with an Accelerometer Cut Point to Classify Physical Activity among Pregnant Obese Women.
PLoS ONE, 2012. isabelle.marc@crchul.ulaval.ca

Traduit et adapté de l'anglais (Chasan-Taber L *et al*. Med Sci Sports Exerc. 2004 Oct;36(10):1750-60)

Il est très important que vous répondiez honnêtement aux questions. Il n'y a pas de bonne ou de mauvaise réponse. Nous voulons seulement connaître les choses que vous avez faites dans les trois (3) derniers mois.

1. Date d'aujourd'hui:
___/___/___/___ ___/___ ___/___
Année Mois Jour

2. Quelle est la date du premier jour de vos dernières menstruations?
___/___/___/___ ___/___ ___/___ ☐ Je ne sais pas
Année Mois Jour

3. Quelle est la date prévue d'accouchement?
___/___/___/___ ___/___ ___/___ ☐ Je ne sais pas
Année Mois Jour

Dans les trois (3) derniers mois, quand vous N'étiez PAS au travail, combien de temps passiez-vous généralement à :

4. Préparer les repas (cuisiner, mettre la table, laver la vaisselle)
   - ☐ Jamais
   - ☐ Moins de 1/2h / jour
   - ☐ 1/2h à presque 1h / jour
   - ☐ 1h à presque 2h / jour
   - ☐ 2h à presque 3h / jour
   - ☐ 3h ou plus / jour

5. Habiller, laver et nourrir les enfants en étant <u>assise</u>
   - ☐ Jamais
   - ☐ Moins de 1/2h / jour
   - ☐ 1/2h à presque 1h / jour
   - ☐ 1h à presque 2h / jour
   - ☐ 2h à presque 3h / jour
   - ☐ 3h ou plus / jour

6. Habiller, laver et nourrir les enfants en étant <u>debout</u>
   - ☐ Jamais
   - ☐ Moins de 1/2h / jour
   - ☐ 1/2h à presque 1h / jour
   - ☐ 1h à presque 2h / jour
   - ☐ 2h à presque 3h / jour
   - ☐ 3h ou plus / jour

7. Jouer avec les enfants en étant <u>assise ou debout</u>
   - ☐ Jamais
   - ☐ Moins de 1/2h / jour
   - ☐ 1/2h à presque 1h / jour
   - ☐ 1h à presque 2h / jour
   - ☐ 2h à presque 3h / jour
   - ☐ 3h ou plus / jour

8. Jouer avec les enfants en <u>marchant ou courant</u>
   - ☐ Jamais
   - ☐ Moins de 1/2h / jour
   - ☐ 1/2h à presque 1h / jour
   - ☐ 1h à presque 2h / jour
   - ☐ 2h à presque 3h / jour
   - ☐ 3h ou plus / jour

9. Porter des enfants (dans les bras, porte-bébé, sur le dos, etc.)
   - ☐ Jamais
   - ☐ Moins de 1/2h / jour
   - ☐ 1/2h à presque 1h / jour
   - ☐ 1h à presque 2h / jour
   - ☐ 2h à presque 3h / jour
   - ☐ 3h ou plus / jour

Dans les trois (3) derniers mois, quand vous N'étiez PAS au travail, combien de temps passiez-vous généralement à :

10. Prendre soin d'une personne âgée
    - ☐ Jamais
    - ☐ Moins de 1/2h / jour
    - ☐ 1/2h à presque 1h / jour
    - ☐ 1h à presque 2h / jour
    - ☐ 2h à presque 3h / jour
    - ☐ 3h ou plus / jour

11. Vous asseoir pour utiliser un ordinateur ou écrire, lorsque vous n'êtes pas au travail
    - ☐ Jamais
    - ☐ Moins de 1/2h / jour
    - ☐ 1/2h à presque 1h / jour
    - ☐ 1h à presque 2h / jour
    - ☐ 2h à presque 3h / jour
    - ☐ 3h ou plus / jour

12. Regarder la télévision, une vidéo ou un DVD
    - ☐ Jamais
    - ☐ Moins de 1/2h / jour
    - ☐ 1/2h à presque 2h / jour
    - ☐ 2h à presque 4h / jour
    - ☐ 4h à presque 6h / jour
    - ☐ 6h ou plus / jour

13. Vous asseoir pour lire, parler, ou téléphoner, lorsque vous n'êtes pas au travail
    - ☐ Jamais
    - ☐ Moins de 1/2h / jour
    - ☐ 1/2h à presque 2h / jour
    - ☐ 2h à presque 4h / jour
    - ☐ 4h à presque 6h / jour
    - ☐ 6h ou plus / jour

14. Jouer avec des animaux domestiques
    - ☐ Jamais
    - ☐ Moins de 1/2h / jour
    - ☐ 1/2h à presque 1h / jour
    - ☐ 1h à presque 2h / jour
    - ☐ 2h à presque 3h / jour
    - ☐ 3h ou plus / jour

15. Faire les tâches ménagères habituelles (faire les lits, faire la lessive, repasser, ranger les choses)
    - ☐ Jamais
    - ☐ Moins de 1/2h / jour
    - ☐ 1/2h à presque 1h / jour
    - ☐ 1h à presque 2h / jour
    - ☐ 2h à presque 3h / jour
    - ☐ 3h ou plus / jour

16. Magasiner (nourriture, vêtements, autres items)
    - ☐ Jamais
    - ☐ Moins de 1/2h / jour
    - ☐ 1/2h à presque 1h / jour
    - ☐ 1h à presque 2h / jour
    - ☐ 2h à presque 3h / jour
    - ☐ 3h ou plus / jour

17. Faire le ménage (passer l'aspirateur, passer la vadrouille, balayer, laver les fenêtres)
    - ☐ Jamais
    - ☐ Moins de 1/2h / semaine
    - ☐ 1/2h à presque 1h/semaine
    - ☐ 1h à presque 2h / semaine
    - ☐ 2h à presque 3h / semaine
    - ☐ 3h ou plus par semaine

18. Tondre la pelouse à l'aide d'un tracteur à pelouse (position assise)
    - ☐ Jamais
    - ☐ Moins de 1/2h / semaine
    - ☐ 1/2h à presque 1h/semaine
    - ☐ 1h à presque 2h / semaine
    - ☐ 2h à presque 3h / semaine
    - ☐ 3h ou plus / semaine

19. Tondre la pelouse à l'aide d'une tondeuse à gazon (debout), râteler les feuilles, jardiner, pelleter la neige
    - ☐ Jamais
    - ☐ Moins de 1/2h / semaine
    - ☐ 1/2h à presque 1h/semaine
    - ☐ 1h à presque 2h / semaine
    - ☐ 2h à presque 3h / semaine
    - ☐ 3h ou plus / semaine

Se déplacer d'un endroit à l'autre…

Dans les trois (3) derniers mois, combien de temps passiez-vous généralement à :

20. Marcher <u>lentement</u> pour vous déplacer à un endroit (par exemple : pour prendre l'autobus, aller au travail, rendre visite) *<u>Pas</u> pour le plaisir ou l'exercice*
    - ☐ Jamais
    - ☐ Moins de 1/2h / jour
    - ☐ 1/2h à presque 1h / jour
    - ☐ 1h à presque 2h / jour
    - ☐ 2h à presque 3h / jour
    - ☐ 3h ou plus / jour

21. Marcher <u>rapidement</u> pour vous déplacer à un endroit (par exemple : pour prendre l'autobus, aller au travail ou à l'école) *<u>Pas</u> pour le plaisir ou l'exercice*
    - ☐ Jamais
    - ☐ Moins de 1/2h / jour
    - ☐ 1/2h à presque 1h / jour
    - ☐ 1h à presque 2h / jour
    - ☐ 2h à presque 3h / jour
    - ☐ 3h ou plus / jour

22. Conduire ou prendre place dans une voiture ou un autobus
    - ☐ Jamais
    - ☐ Moins de 1/2h / jour
    - ☐ 1/2h à presque 1h / jour
    - ☐ 1h à presque 2h / jour
    - ☐ 2h à presque 3h / jour
    - ☐ 3h ou plus / jour

Pour le plaisir ou comme exercice…

Dans les trois (3) derniers mois, combien de temps passiez-vous généralement à :

23. Marcher <u>lentement</u> pour le plaisir ou comme exercice
    - ☐ Jamais
    - ☐ Moins de 1/2h / semaine
    - ☐ 1/2h à presque 1h/semaine
    - ☐ 1h à presque 2h / semaine
    - ☐ 2h à presque 3h / semaine
    - ☐ 3h ou plus / semaine

24. Marcher <u>rapidement</u> pour le plaisir ou comme exercice
    - ☐ Jamais
    - ☐ Moins de 1/2h / semaine
    - ☐ 1/2h à presque 1h/semaine
    - ☐ 1h à presque 2h / semaine
    - ☐ 2h à presque 3h / semaine
    - ☐ 3h ou plus / semaine

25. Marcher <u>rapidement en montée</u> pour le plaisir ou comme exercice
    - ☐ Jamais
    - ☐ Moins de 1/2h / semaine
    - ☐ 1/2h à presque 1h/semaine
    - ☐ 1h à presque 2h / semaine
    - ☐ 2h à presque 3h / semaine
    - ☐ 3h ou plus / semaine

26. Jogger
    - ☐ Jamais
    - ☐ Moins de 1/2h / semaine
    - ☐ 1/2h à presque 1h/semaine
    - ☐ 1h à presque 2h / semaine
    - ☐ 2h à presque 3h / semaine
    - ☐ 3h ou plus / semaine

27. Suivre des cours d'exercices prénataux
    - ☐ Jamais
    - ☐ Moins de 1/2h / semaine
    - ☐ 1/2h à presque 1h/semaine
    - ☐ 1h à presque 2h / semaine
    - ☐ 2h à presque 3h / semaine
    - ☐ 3h ou plus / semaine

28. Nager
    - ☐ Jamais
    - ☐ Moins de 1/2h / semaine
    - ☐ 1/2h à presque 1h/semaine
    - ☐ 1h à presque 2h / semaine
    - ☐ 2h à presque 3h / semaine
    - ☐ 3h ou plus / semaine

29. Danser
    - ☐ Jamais
    - ☐ Moins de 1/2h / semaine
    - ☐ 1/2h à presque 1h/semaine
    - ☐ 1h à presque 2h / semaine
    - ☐ 2h à presque 3h / semaine
    - ☐ 3h ou plus / semaine

Faites-vous autre(s) chose(s) pour le plaisir ou comme exercice? S'il-vous-plaît, nommez-les.

30. ______________________________
    Nom de l'activité
    - ☐ Jamais
    - ☐ Moins de 1/2h / semaine
    - ☐ 1/2h à presque 1h/semaine
    - ☐ 1h à presque 2h / semaine
    - ☐ 2h à presque 3h / semaine
    - ☐ 3h ou plus / semaine

31. ______________________________
    Nom de l'activité
    - ☐ Jamais
    - ☐ Moins de 1/2h / semaine
    - ☐ 1/2h à presque 1h/semaine
    - ☐ 1h à presque 2h / semaine
    - ☐ 2h à presque 3h / semaine
    - ☐ 3h ou plus / semaine

Au travail…

S'il vous plait, complétez la prochaine section si vous travaillez avec rémunération, comme bénévole ou si vous êtes une étudiante. Si vous êtes au foyer, en retrait préventif à la maison, sans emploi ou inapte au travail, vous n'avez pas besoin de remplir cette dernière section.

Dans les trois (3) derniers mois, combien de temps passiez-vous généralement à :

32. Être assise pendant le travail ou en classe
    - ☐ Jamais
    - ☐ Moins de 1/2h / jour
    - ☐ 1/2h à presque 2h / jour
    - ☐ 2h à presque 4h / jour
    - ☐ 4h à presque 6h / jour
    - ☐ 6h ou plus / jour

33. Être debout ou marcher lentement pendant le travail tout en transportant des choses plus lourdes qu'un gallon [4 litres] de lait
    - ☐ Jamais
    - ☐ Moins de 1/2h / jour
    - ☐ 1/2h à presque 2h / jour
    - ☐ 2h à presque 4h / jour
    - ☐ 4h à presque 6h / jour
    - ☐ 6h ou plus / jour

34. Être debout ou marcher lentement pendant le travail sans transporter quoi que ce soit
    - ☐ Jamais
    - ☐ Moins de 1/2h / jour
    - ☐ 1/2h à presque 2h / jour
    - ☐ 2h à presque 4h / jour
    - ☐ 4h à presque 6h / jour
    - ☐ 6h ou plus / jour

35. Marcher rapidement pendant le travail tout en transportant des choses plus lourdes qu'un gallon [4 litres] de lait
    - ☐ Jamais
    - ☐ Moins de 1/2 heure / jour
    - ☐ 1/2h à presque 2h / jour
    - ☐ 2h à presque 4h / jour
    - ☐ 4h à presque 6h / jour
    - ☐ 6h ou plus / jour

36. Marcher rapidement pendant le travail sans transporter quoi que ce soit
    - ☐ Jamais
    - ☐ Moins de 1/2h / jour
    - ☐ 1/2h à presque 2h / jour
    - ☐ 2h à presque 4h / jour
    - ☐ 4h à presque 6h / jour
    - ☐ 6h ou plus / jour

Merci
