# Supplementary material for: French Pregnancy Physical Activity Questionnaire Compared with an Accelerometer Cut Point to Classify Physical Activity among Pregnant Obese Women
Source: PLoS One. 2012 Jun 11;7(6):e38818. doi: 10.1371/journal.pone.0038818 (PMC3372468; doi:10.1371/journal.pone.0038818)
Supplement: File S2 — Physical activity distribution during pregnancy from Actigraph’s GT1M recording in pregnant obese women (Hendelman’s, Swartz’s and Freedson’s cut points). (PDF) [file pone.0038818.s002.pdf]

File S2: Physical activity distribution during pregnancy from Actigraph's GT1M recording in pregnant obese women (Hendelman's, Swartz's and Freedson's cut points).

	All participants (*n*=48)			First trimester (*n*=17)	Second trimester (*n*=16)	Third trimester (*n*=15)
	Mean ± SD or *n* (%)	Median	25th-75th percentile	Mean ± SD or *n* (%)	Mean ± SD or *n* (%)	Mean ± SD or *n* (%)
Moderate intensity or above (min.24h^{-1})						
Hendelman's cut point	270 ± 76	269	228 – 300	264 ± 55	273 ± 75	273 ± 98
Swartz's cut point	119 ± 44	107	89 – 149	121 ± 30	115 ± 45	122 ± 58
Freedson's cut point	14 ± 10	12	5 – 22	20 ± 10	10 ± 7	11 ± 8
Cumulating 150 min of moderate intensity activity by week						
Hendelman's cut point	48 (100%)			17 (100%)	16 (100%)	15 (100%)
Swartz's cut point	48 (100%)			17 (100%)	16 (100%)	15 (100%)
Freedson's cut point	12 (25%)			9 (53%)	1 (6%)	2 (13%)
