# Supplementary material for: French Pregnancy Physical Activity Questionnaire Compared with an Accelerometer Cut Point to Classify Physical Activity among Pregnant Obese Women
Source: PLoS One. 2012 Jun 11;7(6):e38818. doi: 10.1371/journal.pone.0038818 (PMC3372468; doi:10.1371/journal.pone.0038818)
Supplement: File S3 — Accuracy of the French PPAQ in pregnant obese women (Hendelman’s, Swartz’s and Freedson’s cut points). (PDF) [file pone.0038818.s003.pdf]

File S3: Accuracy of the French PPAQ in pregnant obese women (Hendelman's, Swartz's and Freedson's cut points).

Methods: Relationships between activity and Actigraph GT1M (criterion) data (Spearman correlation coefficients [SCCs])

Summary Results	Hendelman's cut point	Swartz's cut point	Freedson's cut point
Total activity (light and above)	**0.67 (*p*<0.01)**	**0.56 (*p*<0.01)**	**0.29 (*p*<0.05)**
Sedentary (<2.0 METs)	**-0.38 (*p*<0.01)**	-0.23 (*p*=0.11)	0.01 (*p*=0.94)
Light (2.0 – <3.0 METs)	**0.68 (*p*<0.01)**	**0.54 (*p*<0.01)**	0.22 (*p*=0.13)
Moderate (3.0 – 6.0 METs)	**0.50 (*p*<0.01)**	**0.44 (*p*<0.01)**	0.22 (*p*=0.14)
Vigorous (>6.0 METs)	**0.32 (*p*=0.03)**	0.28 (*P*=0.06)	**0.29 (*p*<0.05)**
Household/Caregiving	**0.68 (*p*<0.01)**	**0.55 (*p*<0.01)**	0.20 (*p*=0.18)
Occupational (*n*=19)*	**0.64 (*p*<0.01)**	**0.61 (*p*<0.01)**	0.20 (*p*=0.42)
Sports/Exercises	**0.36 (*p*=0.01)**	**0.32 (*p*=0.03)**	**0.30 (*p*=0.04)**
Transportation	**0.35 (*p*=0.02)**	0.28 (*P*>0.05)	**0.38 (*p*<0.01)**

* Including only women who were still working in the past trimester
