# Supplementary material for: French Pregnancy Physical Activity Questionnaire Compared with an Accelerometer Cut Point to Classify Physical Activity among Pregnant Obese Women
Source: PLoS One. 2012 Jun 11;7(6):e38818. doi: 10.1371/journal.pone.0038818 (PMC3372468; doi:10.1371/journal.pone.0038818)
Supplement: File S4 — GT1M values across tertiles of total energy expenditure based on the French PPAQ in pregnant obese women (Hendelman’s, Swartz’s and Freedson’s cut points). (PDF) [file pone.0038818.s004.pdf]

File S4: GT1M values across tertiles of total energy expenditure based on the French PPAQ in pregnant obese women (Hendelman's, Swartz's and Freedson's cut points).

Actigraph measures	Lowest Tertile	Middle Tertile	Highest Tertile	Trend *p**
	Mean ±SD (*n*=15)	Mean ±SD (*n*=18)	Mean ±SD (*n*=15)	
Hendelman's cut point	238 ± 68	254 ± 70	320 ± 67	**<0.01**
Swartz's cut point	104 ± 39	113 ± 37	143 ± 49	**0.02**
Freedson's cut point	10 ± 8	14 ± 9	17 ± 11	0.09

*Jonckheere-Terpstra
