# Supplementary material for: French Pregnancy Physical Activity Questionnaire Compared with an Accelerometer Cut Point to Classify Physical Activity among Pregnant Obese Women
Source: PLoS One. 2012 Jun 11;7(6):e38818. doi: 10.1371/journal.pone.0038818 (PMC3372468; doi:10.1371/journal.pone.0038818)
Supplement: File S5 — Physical activity distribution during pregnancy from Actigraph’s GT1M recording in pregnant obese women (Bouts of at least 10 consecutive minutes over standard cut points). (PDF) [file pone.0038818.s005.pdf]

File S5: Physical activity distribution during pregnancy from Actigraph's GT1M recording in pregnant obese women (Bouts of at least 10 consecutive minutes over standard cut points).

	All participants (*n*=48)			First trimester (*n*=17)	Second trimester (*n*=16)	Third trimester (*n*=15)
	Mean ± SD or *n* (%)	Median	25th-75th percentile	Mean ± SD or *n* (%)	Mean ± SD or *n* (%)	Mean ± SD or *n* (%)
Moderate intensity or above (min.24h^{-1})						
Hendelman's	124 ± 69	114	76 – 163	120 ± 40	123 ± 76	129 ± 89
Swartz's	28 ± 25	18	8 – 45	33 ± 20	21 ± 23	30 ± 32
Matthews's	17 ± 16	10	4 – 31	24 ± 15	11 ± 15	16 ± 16
Freedson's	5 ± 7	0	0 – 8	10 ± 8	1 ± 2	2 ± 4
Cumulating 150 min of moderate intensity activity by week						
Hendelman's	48 (100%)			17 (100%)	16 (100%)	15 (100%)
Swartz's	23 (48%)			11 (65%)	6 (38%)	6 (40%)
Matthews's	16 (33%)			9 (53%)	2 (13%)	5 (33%)
Freedson's	12 (25%)			9 (53%)	1 (6%)	2 (13%)
