# Supplementary material for: French Pregnancy Physical Activity Questionnaire Compared with an Accelerometer Cut Point to Classify Physical Activity among Pregnant Obese Women
Source: PLoS One. 2012 Jun 11;7(6):e38818. doi: 10.1371/journal.pone.0038818 (PMC3372468; doi:10.1371/journal.pone.0038818)
Supplement: File S6 — Accuracy of the French PPAQ in pregnant obese women (Bouts of at least 10 consecutive minutes over standard cut points). (PDF) [file pone.0038818.s006.pdf]

File S6: Accuracy of the French PPAQ in pregnant obese women (Bouts of at least 10 consecutive minutes over standard cut points).

Methods: Relationships between activity and Actigraph GT1M (criterion) data (Spearman correlation coefficients [SCCs])

Summary Results	Hendelman's cut point	Swartz's cut point	Matthews's cut point	Freedson's cut point
Total activity (light and above)	**0.53 (*p*<0.01)**	**0.42(*p*<0.01)**	**0.35 (*p*=0.02)**	0.19 (*p*=0.20)
Sedentary (<2.0 METs)	-0.20 (*p*=0.17)	-0.07 (*p*=0.61)	-0.07 (*p*=0.65)	0.12 (*p*=0.43)
Light (2.0 – <3.0 METs)	**0.53 (*p*<0.01)**	**0.37 (*p*<0.01)**	**0.29 (*p*=0.04)**	0.14 (*p*=0.33)
Moderate (3.0 – 6.0 METs)	**0.37 (*p*=0.01)**	**0.33 (*p*=0.02)**	0.27 (*p*=0.07)	0.14 (*p*=0.33)
Vigorous (>6.0 METs)	0.27 (*p*=0.07)	**0.29 (*p*=0.04)**	**0.30 (*p*=0.04)**	0.23 (*p*=0.12)
Household/Caregiving	**0.52 (*p*<0.01)**	**0.39 (*p*<0.01)**	**0.31 (*p*=0.03)**	0.10 (*p*=0.51)
Occupational (*n*=19)*	**0.50 (*p*=0.03)**	0.27 (*p*=0.26)	0.19 (*p*=0.44)	0.11 (*p*=0.66)
Sports/Exercises	**0.29 (*p*<0.05)**	0.26 (*p*=0.08)	0.24 (*p*=0.10)	0.20 (*p*=0.18)
Transportation	0.25 (*p*=0.09)	**0.32 (*p*=0.02)**	**0.38 (*p*<0.01)**	**0.33 (*p*=0.02)**

* Including only women who were still working in the past trimester
