# Supplementary material for: French Pregnancy Physical Activity Questionnaire Compared with an Accelerometer Cut Point to Classify Physical Activity among Pregnant Obese Women
Source: PLoS One. 2012 Jun 11;7(6):e38818. doi: 10.1371/journal.pone.0038818 (PMC3372468; doi:10.1371/journal.pone.0038818)
Supplement: File S7 — GT1M values across tertiles of total energy expenditure based on the French PPAQ in pregnant obese women (Bouts of at least 10 consecutive minutes over standard cut points). (PDF) [file pone.0038818.s007.pdf]

File S7: GT1M values across tertiles of total energy expenditure based on the French PPAQ in pregnant obese women (Bouts of at least 10 consecutive minutes over standard cut points)

Actigraph measures	Lowest Tertile	Middle Tertile	Highest Tertile	Trend *p* [*]
	Mean±SD (*n*=15)	Mean±SD (*n*=18)	Mean±SD (*n*=15)	
Hendelman's cut point	101 ± 61	110 ± 56	164 ± 78	**0.02**
Swartz's cut point	21 ± 20	23 ± 18	42 ± 32	**0.04**
Matthews's cut point	13 ± 13	15 ± 13	25 ± 20	0.10
Freedson's cut point	3 ± 5	5 ± 7	6 ± 7	0.18

[*] Jonckheere-Terpstra
